# A novel subgroup Q5 of human Y-chromosomal haplogroup Q in India

**DOI:** 10.1186/1471-2148-7-232

**Published:** 2007-11-19

**Authors:** Swarkar Sharma, Ekta Rai, Audesh K Bhat, Amarjit S Bhanwer, Rameshwar NK Bamezai

**Affiliations:** 1 National Centre of Applied Human Genetics, School of Life Sciences, Jawaharlal Nehru University, New Delhi. India; 2 Department of Human Genetics, Guru Nanak Dev University, Amritsar. India

## Abstract

**Background:**

Y-chromosomal haplogroup (Y-HG) Q is suggested to originate in Asia and represent recent founder paternal Native American radiation into the Americas. This group is delineated into Q1, Q2 and Q3 subgroups defined by biallelic markers M120, M25/M143 and M3, respectively. Recently, a novel subgroup Q4 has been identified which is defined by bi-allelic marker M346, representing HG Q (0.41%, 3/728) in Indian population. With scanty details of HG Q in Asia, especially India, it was pertinent to explore the status of the Y-HG Q in Indian population to gather an insight to determine the extent of diversity within this region.

**Results:**

We observed 15/630 (2.38%) Y-HG Q individuals in India with an ancestral state at M120, M25, M3 and M346 markers, indicating an absence of already known Q1, Q2, Q3 and Q4 sub-haplogroups. Interestingly, we further observed a novel 4 bp deletion/insertion polymorphism (ss4 bp, rs41352448) at 72,314 position of human arylsulfatase D pseudogene, defining a novel sub-lineage Q5 (in 5/15 individuals, i.e., 33.3 % of the observed Y-HG Q) with distributions independent of the social, cultural, linguistic and geographical affiliations in India.

**Conclusion:**

The study adds another sublineage Q5 in the already existing arrangement of Y-HG Q in literature. It was quite interesting to observe an ancestral state Q* and a novel sub-branch Q5, not reported elsewhere, in Indian subcontinent, though in low frequency. A novel subgroup Q4 was identified recently which is also restricted to Indian subcontinent. The most plausible explanation for these observations could be an ancestral migration of individuals bearing ancestral lineage Q* to Indian subcontinent followed by an autochthonous differentiation to Q4 and Q5 sublineages later on. However, other explanations of, either the presence of both the sub haplogroups (Q4 and Q5) in ancestral migrants or recent migrations from central Asia, cannot be ruled out till the distribution and diversity of these subgroups is explored extensively in Central Asia and other regions.

## Background

In the past, markers on the non-recombining region of the Y chromosome (NRY) have been used extensively as a male complement to mtDNA to study the colonization or migration histories within the different regions of the world. Y-chromosomal haplogroup (Y-HG) Q, defined by either of the binary markers P36/MEH2 [1] or M242 [2], has been suggested to originate in Asia and represent recent founder paternal Native American radiation into the Americas. In Eurasia, haplogroup Q chromosomes have been reported with the highest frequency in Siberian populations, distributed primarily across Northwest and Northeast Siberia with the vast majority in only two Siberian populations, the Kets (93.8%) and the Selkups (66.4%) [3]. Other population groups from the region bearing this haplogroup include, Kyrgyz (2%), Kazak (6%), Kallar (1%), Shiraz (8%), Bartangi (13%), Korean (2%), Yagnobi (3%), Esfahan (6%), Turkmen (10%), Dungan (8%), Tuvinian (17%), Uzbek/Kashkadarya (5%), Shugnan (11%), Uzbek/Bukhara (2%), Uzbek/Surkhandarya (4%), Yadhava (3%), Kazan Tatar (3%), Tajik/Samarkand (5%), Uighur (5%), Uzbek/Khorezm (9%), Uzbek/Tashkent (14%), Arab/Bukhara (14%), Uzbek/Fergana Valley (5%) and Uzbek/Samarkand 7%) [2]. Interestingly, Y-HG Q is the youngest paternal haplogroup observed with less frequent subgroups which are geographically restricted. These have been delineated into Q1, Q2 and Q3 subgroups defined by biallelic markers M120, M25/M143 and M3, respectively[1]. Recently, a novel subgroup Q4 was identified, defined by the bi-allelic marker M346, representing HG Q (0.41%, 3/728) in Indian populations [4].

With very less details of HG Q in Asia, especially India, it was pertinent to explore the status of the Y-HG Q in Indian populations to gather an insight to determine the extent of diversity within this region.

## Results and Discussion

In the present study, we screened 630 samples belonging to different regions of India and observed 2.38% (15/630) individuals bearing Y-HG Q. It was interesting to observe that 14/15 samples did not show any of the already known Y-HG Q sub-haplogroups (Q1, Q2, Q3 and Q4), defined by biallelic markers M120, M25/M143, M3 and M346, respectively (Table 1). Only one individual was observed with the presence of M120 polymorphism, representing Q1 lineage.

**Table 1:** Indian populations screened for Y-HG Q and its distribution.

**India Regions**	**Social Category**		**Linguistic Category**	**No. of Samples**		**No. of Samples in Haplogroup**	
**North:**						Q (xQ5)	Q5
J&K Kashmiri Pandits	Caste	high	Indo-European	51	*	3	
J&K kashmir Gujars	Tribe		Indo-European	61	*	1^Q1^	
Dalits^+^	Caste	low	Indo-European	18	^#^		
Muslim	Religious	group	Indo-European	19	^#^		
Rajput	Caste	high	Indo-European	29	^#^		
Uttar Pradesh Brahmin	Caste	high	Indo-European	14	^#^	1^@^	
Uttar Pradesh Brahmin	Caste	high	Indo-European	31	*	1	1
Uttar Pradesh Mixed	Tribe		Austro-Asiatic	9	*		
Punjab Brahmin	Caste	high	Indo-European	28	*		
Himachal Brahmin	Caste	high	Indo-European	19	*		
Himachal Rajputs	Caste	high	Indo-European	35	*	1	
							
**Central:**							
Uttar Pradesh Kols	Tribe		Indo-European/Austro-Asiatic	30	*		
Uttar Pradesh gonds	Tribe		Indo-European/Dravidian	38	*		
Madhya Pradesh Brahmins	Caste	high	Indo-European	42	*	1	1
Madhya Pradesh Gonds	Tribe		Dravidian	17	*	1	
Madhya Pradesh Saharia	Tribe		Indo-European	89	*	1	^2^
Halba	Tribe		Indo-European	21	^#^	1^@^	
Kamar	Tribe		Dravidian	30	^#^		
Muria	Tribe		Dravidian	20	^#^		
							
**East:**							
Bihar Brahmins	Caste	high	Indo-European	38	*	1	1
Paswan	Caste	low	Indo-European	29	*		
Agharia	Caste	middle	Indo-European	10	^#^		
Bagdi	Caste	low	Indo-European	11	^#^		
Gaud	Caste	middle	Indo-European	5	^#^		
Ho	Tribe		Austro-Asiatic	30	^#^		
Lodha	Tribe		Austro-Asiatic	20	^#^		
Mahishya	Caste	middle	Indo-European	13	^#^		
Santal	Tribe		Austro-Asiatic	14	^#^		
Tanti	Caste	low	Indo-European	7	^#^		
West Bengal Brahmin	Caste	high	Indo-European	18	^#^		
							
**Northeast:**							
Chakma	Tribe		Tibeto-Burman	4	^#^		
Jamatia	Tribe		Tibeto-Burman	30	^#^		
Mog	Tribe		Tibeto-Burman	5	^#^		
Mizo	Tribe		Tibeto-Burman	27	^#^		
Tripuri	Tribe		Tibeto-Burman	21	^#^		
							
**West:**							
Maharashtra Brahmins	Caste	high	Indo-European	30	*		
Gujarat Bhils	Tribe		Indo-European	12	*		
Gujarat Brahmins	Caste	high	Indo-European	63	*		
Sourashtran			Indo-European	46	^$^		
Koknasth Brahmin	Caste	high	Indo-European	25	^#^		
Maratha	Caste	middle	Indo-European	20	^#^		
Nav Buddha	Caste	low	Indo-European	14	^#^		
							
**South:**							
Andhra Brahmins	Caste	high	Dravidian	6	*		
Kallar	Caste		Dravidian	84	^$^		
Yadhava	Caste		Dravidian	129	^$^	3	
Ambalakarar	Caste	middle	Dravidian	29	^#^		
Irula	Tribe		Dravidian	30	^#^		
Iyengar	Caste	high	Dravidian	30	^#^		
Iyer	Caste	high	Dravidian	29	^#^		
Koya	Tribe	Tribe	Dravidian	27	^#^		
Kota	Tribe		Dravidian	16	^#^		
Konda	Tribe	Tribe	Dravidian	30	^#^		
Kurumba	Tribe		Dravidian	19	^#^		
Pallan	Caste	low	Dravidian	29	^#^		
Toda	Tribe		Dravidian	8	^#^		
Vanniyar	Caste	middle	Dravidian	25	^#^		
Vellalar	Caste	middle	Dravidian	31	^#^	1^@^	

**Total**				**1615**		**16**	**5**

* Present Study, # Sengupta et al. 2006, $ Seielstad et al. 2003.

'Dalits^+^' used instead of 'Chamar', as per Indian Constitution.

^@^found as 'Q4' by Sengupta et al.^4^

^Q1^Found as Q1 in present study.

**Figure 1:**
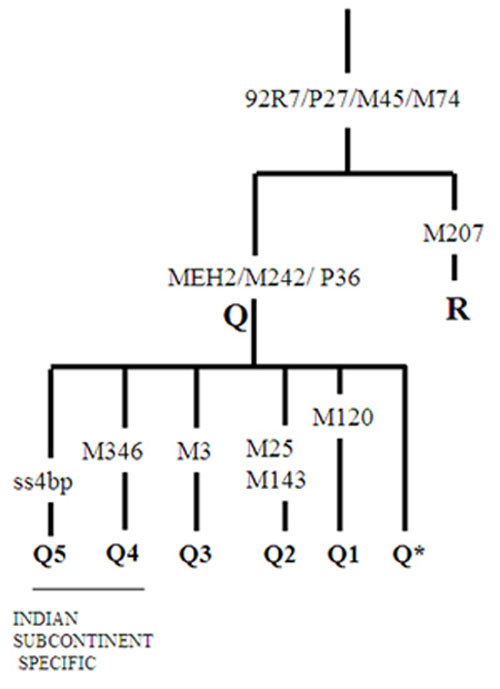
Partial YCC tree redrawn with the addition of M346 marker defining Y-HG Q4 and ss4 bp marker defining Y-HG Q5.

Further, a novel 4 bp del/ins polymorphism (rs41352448, details provided in the Additional file 1) at 72,314 position of human arylsulfatase D pseudogene (ARSDP gene), in 5/15 individuals (33.3% of the observed Y-HG Q in the study) indicated the presence of a novel sub-group Q5 of Y-HG Q. In order to establish the exclusiveness of this polymorphism to Y-HG Q, we screened some of our samples already categorized in other unrelated haplogroups like R1a1, R2, L, H1, J, C, etc. The presence of an ancestral allele (without insertion at 72,314 position of ARSDP gene) in these samples confirmed the restriction of this novel polymorphism within the Y-HG Q. We also screened the sample with a derived state at M120 marker in the present study (representing Q1), using ss4 bp marker and found an absence of the polymorphism. In order to assign an independent status to designated Q5 and to confirm the placement of M346 derived samples as Q4 [4], it was necessary to study the novel ss4 bp marker (Q5) in M346 derived samples (Table 1). Three samples provided on request were screened for the ss4 bp polymorphism. The absence of this polymorphism in these three samples not only confirmed the authenticity of Q4 lineage but also validated the independent status of Q5 observed by us (Figure 1). In addition to the Y-HG Q5 (5/15) and Q1 (1/15) samples, there were 9/15 (60%) individuals who did not show any of the known Q subgroup signature and were designated as Q*.

**Figure 2:**
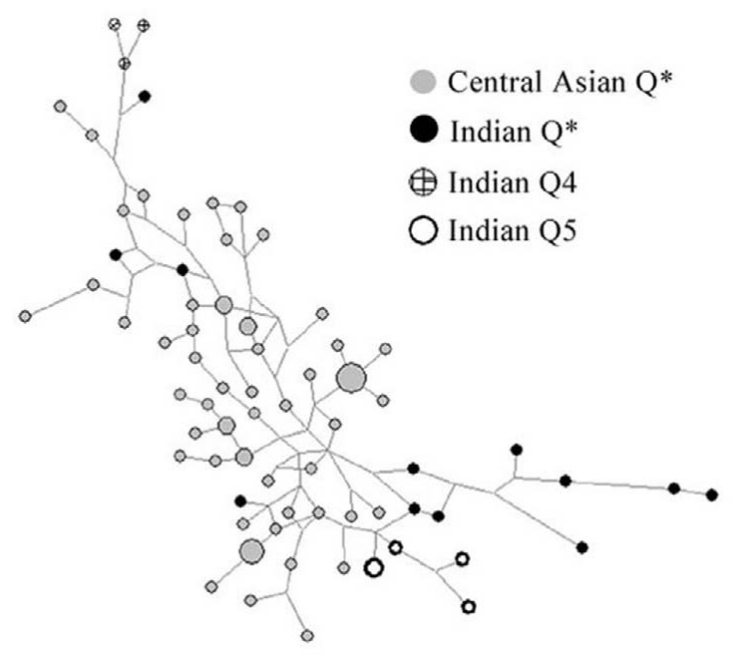
Median joining network-showing relationship of individuals bearing Y-HG Q and its subgroups in population groups of Central Asia and India.

In order to put together our observations along with those made in literature earlier, we pooled data of 1615 Y- chromosomes (630 present study and 985 from literature) (Table 1) for analyses, of which 21/1615 (1.3%) samples represented Q lineage in India. All of our 15 samples and 3 samples belonging to Y-HG Q4 [4] were genotyped for 12 Y-microsatellite markers. However, to keep uniformity in evaluation, data of 10 overlapping Y-STR markers from present study and from different population groups of central Asia and India was used to construct median joining network [5], although 12 Y-STR markers were analysed by us (Additional file 2). For most of the Indian Y-HG Q and its sub-lineages, three clusters of Y-STR haplotypes were observed (Figure 2). One cluster included all the three Q4 and one Q*, another with all the Q5 and the third with most of the Q* bearing individuals. It was interesting to find that most of the Indian Q (Q4, Q5 and Q*) associated Y-STR haplotypes were separated from the bulk of Central Asian Q* associated haplotypes. Further, the clustering of the Y-STR haplotypes reassured the findings by the bi-allelic markers. This could either be due to population differentiation or because of the presence of these clusters in the ancestral migration from Central Asia, not clear at the moment. The increased diversity within the Indian population clusters could be interpreted as an overall effect of geographical differentiation, population expansions and severe bottlenecks resulting in loss of many of the in-between haplotypes thus, reducing the reticulation and increasing the branch lengths. It could also be as a result of independent migrations and admixture.

The age estimations made using a small sample size need to be increased which is not feasible at the moment, keeping in mind a very low frequency of Y-HG Q in India. The age estimation for haplogroup Q in India was carried out on the bigger cluster bearing Q* and Q5 in the median joining network. The calculated age of 47,101.5 (34,210.5 – 75,581.4) Years at 95% CI appears to be an over estimate than the age of haplogroup Q (15,000–18,000 Years Before Present) in literature[2,3,6]. This probably has occurred due to the enhanced diversity, probably as an effect of population expansions and severe bottlenecks or might be due to later migrations and admixture. A further estimation of the age of Y-HG Q5 alone, using similar parameters, provided an age estimate of 14,492.7 (10,526.3 – 23,255.8) Years at 95% CI.

The compilation of distribution pattern of Y-HG Q in Indian population (Table 1) from the present study as well as from literature, points out that this HG is distributed widely, ranging from Indo-European castes and tribes to their Dravidian counterparts, despite its low frequency. These observations could be explained either on the basis of the ancestral relationship of different Indian population groups, irrespective of linguistic and social divisions or alternatively by some degree of recent gene flow between these groups, not clear at the moment.

## Conclusion

Interestingly, apart from assigned Y-HG Q5 (33.3%, 5/15) samples and the only one individual representing Q1 lineage, there were Y-HG Q bearing (60%, 9/15) samples, representing none of the known Q subgroups and designated as Q*. It was quite interesting to observe Y-HG Q in Indian subcontinent though in low frequency but with ancestral Q* state and a novel sub-branch Q5 not reported elsewhere. A novel subgroup Q4 was identified recently which is also restricted to Indian subcontinent [4]. The most plausible explanation for these observations could be an ancestral migration of individuals bearing ancestral lineage Q* to Indian subcontinent with an autochthonous differentiation to Q4 and Q5 sublineages later on. However, other explanations of either the presence of both the sub haplogroups (Q4 and Q5) in ancestral migrants or recent migrations from central Asia, cannot be ruled out till the distribution and diversity of these subgroups is explored extensively in Central Asia and other regions. To conclude, this study presents a novel polymorphism, adding another sublineage Q5 in the already existing arrangement of Y-HG Q in literature.

## Methods

### Samples

We screened 630 Y-chromosomes belonging to various linguistic families, castes and tribes throughout the Indian region (Table 1) to know the status of Y-HG Q and its sublineages and compared it with the data available in literature for different regions of the world [2-4,6,7] and also for Indian population groups [2,4,8].

### Markers and their analysis

The binary markers: M45, 92R7, P36, MEH2, M120, M3, M25 [1], M242 [2] and M346 [4] were used to dissect out known Y-HG Q upto its subgroups. Samples were amplified, checked in 2% agarose gel and then sequenced to detect polymorphisms (using ABI 3100 sequencer, USA). We used primer set [Forward: ttgtccagagaaacagccaat and Reverse: atccatctacctacatacctgtcatc] to define the novel 4 bp (GGAT) deletion/insertion polymorphism 'ss4 bp' [details submitted in the dbSNP (BUILD 127); NCBI Assay ID: ss65713825; refSNP ID: rs41352448 and amplicon sequence provided as Additional file 1] at 72,314 position of human arylsulfatase D pseudogene (ARSDP gene). The total PCR reaction mix made was 12.5 μl, containing 50 ng of template DNA, 6.25 pmoles of each primer, 200 μM of dNTPs, 1.5 mM MgCl_2_, 1× reaction buffer and 0.3 units of *Taq pol *enzyme (Bangalore Genei, India). The cycling conditions were: denaturation at 94°C for 1 min, followed by annealing at 56°C for 1 min, and then extension at 72°C for 1 min, repeated for 30 cycles followed by a final extension step at 72°C for 5 min. PCR products were initially checked in 2% agarose gel, sequenced (using ABI Prism 3100-Avant Genetic Analyzer, Applied Biosystems, USA) and analyzed in SeqScape software v2.1.1 (Applied Biosystems, USA). These samples were also genotyped for 12 Y-microsatellite markers (Y-STRs): DYS388, DYS389I, DYS389II, DYS390, DYS391, DYS392, DYS393, DYS394, DYS426, DYS437, DYS439, DYS448 described elsewhere[9,10] to estimate haplotype variation within the HG defined by binary markers (Additional file 2).

### Statistical and Phylogenetic analysis

Although 12 Y-STR markers were analysed by us (Additional file 2), however, to keep uniformity in evaluation, data of 10 overlapping Y-STR markers from present study and from different population groups of central Asia and India bearing Y-HG Q was used to construct median joining network[5]. Age estimation[11] was carried out using averaged variance (calculated by MICROSAT software, version 1.5d) of the Y-STRs and mutation rate[12] (μ) = 6.9 × 10^-4 ^and 95% CI [9.5 × 10^-4 ^- 4.3 × 10^-4^] per generation (g = 25 years).

## List of abbreviations

arylsulfatase D pseudogene (ARSDP)

Confidence Interval (CI)

Micro litre (μl)

Micro molar (μM)

Y-Chromosome Haplogroup (Y-HG)

Y-Chromosome Short Tandem Repeats (Y-STR)

## Authors' contributions

SS and ER carried out the molecular genetic studies, sequence alignment, statistical and phylogenetic analyses and drafted the manuscript. AKB participated in the sequence alignment and helped in the statistical analysis. AJSB participated in the design of the study. RNKB conceived of the study, and participated in its design, coordination and helped to draft the manuscript. All authors read and approved the final manuscript.

## Supplementary Material

Additional file 1 Full amplicon sequence information of the novel 'ss4 bp' polymorphism. Click here for file

Additional file 2 Number of repeats observed at 12 Y-STRs loci in the individuals with Y-HG Q* and Q5, in the present study. Click here for file
