# Supplementary material for: A novel subgroup Q5 of human Y-chromosomal haplogroup Q in India
Source: BMC Evol Biol. 2007 Nov 19;7:232. doi: 10.1186/1471-2148-7-232 (PMC2258157; doi:10.1186/1471-2148-7-232)
Supplement: Additional file 1 — Full amplicon sequence information of the novel 'ss4 bp' polymorphism. [file 1471-2148-7-232-S1.pdf]

Amplicon sequence of the refSNP ID: **rs41352448** (details in dbSNP (BUILD 127); NCBI Assay ID: ss65713825)

Forward→

5’ttgtccagagaaacagccaataagatgtgtggatgtgtggatggatggatggatggatggatggatggat**ggat**ggaca
gacagctatagaaatacagatagataaaccaatgagtagattatagatagacgagaggagggggagagagaaaaagagaga
gagagaggataatggatatatatagagagagaataatggatagatagatgatagagagatagataaagatacacaatggatgga
tagatatatagataagataaatgataagttacatgatggatggatagatagataagacagatgatggatagatagataatatagata
gatgaaaagtagaagcagttcatggatggatgaatagatggatagatctttcatggatgatggatgaaaagtagatagagatatct
ggtgggctgtttgaaaaagacagatgaccatatagttagataatagatagatgatagacaggtgatagataagttgatcgataaat
agatgatagatggatagaatagatggtagataggtagata**gatgacaggtatgtaggtagatggat 3’**

3’ ctactgtccatacatccatctaccta 5’

←Reverse

Forward primer: 5’ttgtccagagaaacagccaat 3’

Reverse primer: 5’atccatctacctacatacctgtcatc 3’

Q5 subgroup is defined by the insertion of the **ggat** repeat(4bp) at 72,314 position of the human arylsulfatase D pseudogene (ARSDP gene) in individuals with the derived state at the binary markers: M45, 92R7, P36/MEH2/ M242 and ancestral state at the M120, M3, M25/M143 and M346 markers. The hierarchical details of the bi-allelic markers are provided in Figure 1.
